# Combinatorial drug screening of mammary cells with induced mesenchymal transformation to identify drug combinations for triple-negative breast cancer

**DOI:** 10.18632/oncotarget.27104

**Published:** 2019-08-06

**Authors:** Sierra A. Colavito, James T. Platt, Matthew A. Held, Zongzhi Liu, Ryan Sokup, David F. Stern

**Affiliations:** ^1^ Department of Biology, University of Wisconsin-La Crosse, La Crosse, WI, USA; ^2^ Department of Internal Medicine and Yale Cancer Center, Yale School of Medicine, New Haven, CT, USA; ^3^ Department of Molecular, Cellular and Biomedical Sciences, University of New Hampshire, Durham, NH, USA; ^4^ Department of Pathology, Yale School of Medicine, New Haven, CT, USA; ^5^ Yale Cancer Center, New Haven, CT, USA

**Keywords:** epithelial-mesenchymal transition, triple-negative breast cancer, targeted drug combination screen, claudin-low breast cancer, CHK1

## Abstract

Mesenchymal stem-like (MSL) breast cancers are enriched for cells with tumor reconstituting and mesenchymal characteristics. These cancers are often triple-negative and have a poor prognosis. Few effective targeted treatment options exist for patients with these cancers, and even when targeted therapies exist, resistance often arises and tumors recur, due in part to drug-tolerant persisting tumor cells with self-renewal capability. Effective treatment strategies will combine agents that target the bulk-tumor and reconstituting cells. In order to identify such a combination therapy, we conducted an inhibitor screen using 40 targeted agents at three different doses in all pairwise combinations. Checkpoint Kinase 1 (CHK1) inhibitors were identified as potent inhibitors of MSL breast cancers. When combined with a pro-apoptotic agent/B Cell Lymphoma 2 (BCL2) inhibitor, the effectiveness of the combination regimen was super-additive compared to either treatment alone and was selective for MSL cancers. Treatment of MSL breast cancer cells results in DNA damage, cell-cycle defects characterized by a prolonged S-phase, increased apoptosis and decreased colony forming abilities compared to untreated cells. These data suggest that a combination of a CHK1 and BCL2 inhibitor could be an effective treatment for patients with MSL breast cancer. Several other effective drug combinations were also identified.

## INTRODUCTION

Breast cancer is one of the most common and lethal cancers of females in the US and worldwide. There have been significant advances in management of patients with tumors expressing estrogen receptor (ER) or with *HER2/ERBB2* amplification using agents that affect estrogen biosynthesis, or interfere with the ER, or with ERBB2-directed antibodies and tyrosine kinase inhibitors (TKI). However, treatment of triple-negative breast cancers (TNBC) that do not express high levels of HER2/ERBB2, ER, or progesterone receptor (PR) remains a major therapeutic challenge.

Approximately 75% of TNBC are classified through transcriptional subtyping as basal-like breast cancer (BLBC). A less prevalent subset of TNBC are characterized by a Claudin-low (CL) phenotype [1]. In transcriptional comparisons to profiles of normal mammary developmental lineages, BLBC transcriptionally resemble luminal progenitor cells. CL tumors are more enriched for tumor reconstituting cells, and resemble more primitive mammary stem cells. Moreover, these cells have characteristic mesenchymal-like stem-like (MSL) transformation, and are transcriptionally similar to bone marrow-derived mesenchymal stem cells [1]. Similar features are found with MSL transformation induced by genetic manipulation of HMLE mammary cells. HMLE human mammary epithelial cells immortalized with hTERT and SV40 large and small T are enriched for a mammary stem cell/bipotential progenitor phenotype [2]. Epithelial-mesenchymal transition (EMT) induced artificially in HMLE cells through suppression of E-cadherin expression or overexpression of SNAIL greatly enhances stem-like and tumor reconstituting activities, and yields cells with MSL and other features characteristic of CL [3, 4].

TNBC are phenotypically heterogeneous at the cellular level. For example, SUM149PT BLBC cells and HCC38 CL breast cancer (CLBC) cells include subpopulations resembling BLBC and CLBC, with the latter exhibiting faster migration and slower proliferation in culture level [2]. Single cell transcription profiling of human BLBC patient-derived xenografts grown in mice further clarifies the relationship of these phenotypes [5]. Whereas bulk BLBC conform to the BLBC transcriptional pattern, single cell profiling reveals a minority population with MSL features that apparently pioneers metastasis, then repopulates the site with more mature BLBC-like cells.

Overall, these findings suggest that TNBC behavior is consistent with the “cancer stem cell” hypothesis [6–8], whereby minor tumor cell subsets behave as stem/progenitor-like cells and reconstitute a heterogeneous population of cells. Accordingly, therapeutic strategies built upon empirical identification of agents that reduce tumor size will have short term impact, but will fail in the long run if these agents do not eliminate cells that replenish the bulk population post-therapy. Hence, optimal therapies will combine agents that affect the bulk tumor population and the progenitors that likely include MSL cells.

TNBC are often marked by functional activation of the PI3K pathway through multiple mechanisms, and clinical trials are underway to evaluate PI3K inhibitors. Even should some of these inhibitors show acceptable efficacy and tolerability, combination targeting will almost certainly be necessary for enduring responses: driver-targeted cancer therapies (e.g. EGFR targeting for EGFR-driven lung adenocarcinoma and BRAF targeting in BRAF-activated melanoma) yield impressive initial responses, but these responses often fail in under one or two years. Use of combinations of targeted therapies may also help in overcoming the extraordinary genomic complexity of TNBC.

In an earlier study to identify agents that are effective on CL cells and may reduce resilience of BLBC by suppressing MSL drug refuge phenotypic states, we screened 150 single agents for the ability to preferentially affect HMLE cells induced to undergo EMT. We found that induced EMT reduces sensitivity to ERBB inhibitors and increases reliance on NFκB-regulated GLI1 signaling [9]. Here, we have extended this single agent screen to a combinatorial screen for drug combinations that preferentially inhibit growth of HMLE cells with induced EMT. The results identify a number of drug combinations with therapeutic potential, and also revealed that induced EMT cells are hypersensitive to DNA checkpoint kinase inhibitors. These results have implications for therapies for CL and BL TNBC.

## RESULTS

**Figure 1:**
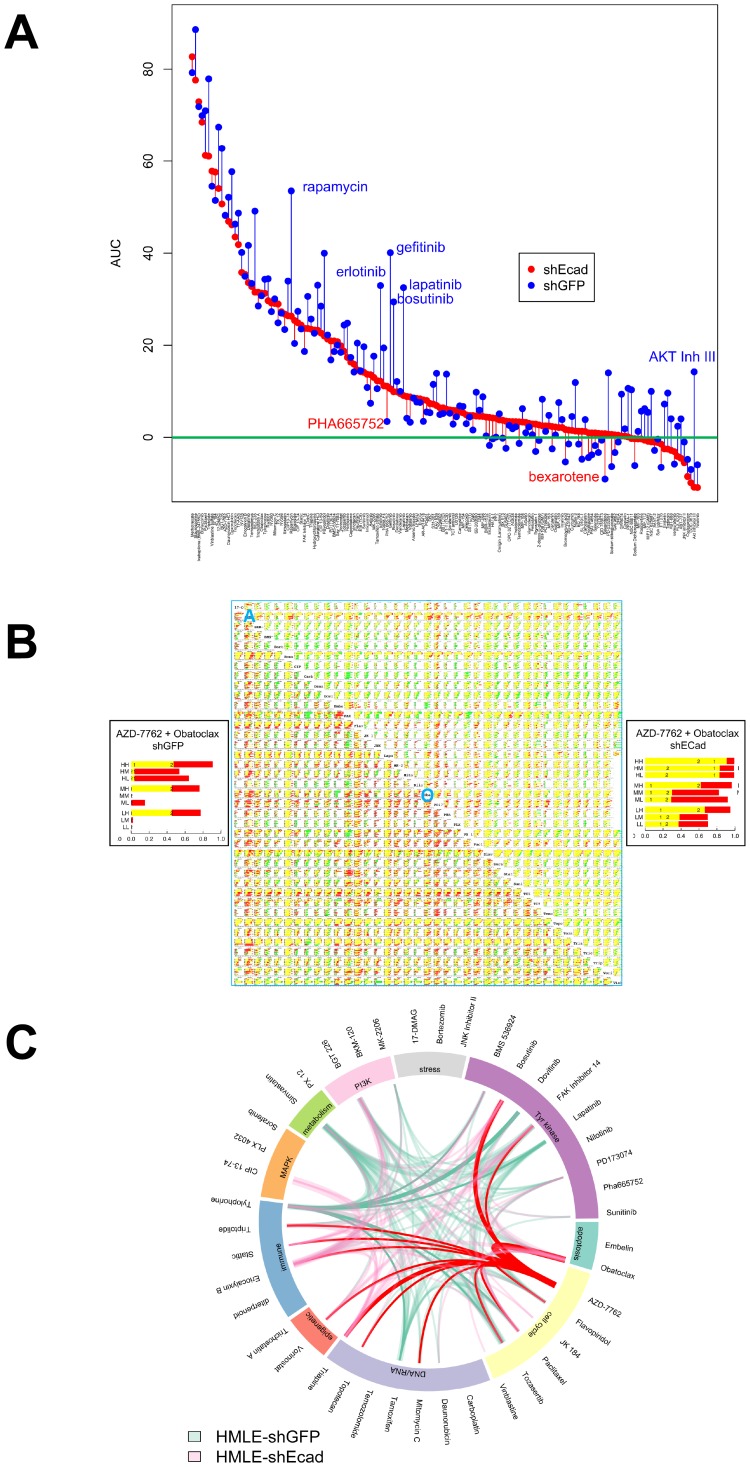
Single agent and combinatorial drug sensitivity of HMLE-shEcad (red) and HMLE-shGFP (blue). (**A**) Plot of AUC average by cell line for each agent. Agents are listed in descending order of AUC of HMLE-shEcad, with AUC plotted for each cell line as marked in legend. For each agent, vertical lines link corresponding AUC of HMLE-shEcad and HMLE-shGFP. These lines are colored red for agents where AUC is greater for HMLE-shECad, and blue for agents for which AUC is greater for HMLE-shGFP. (**B**) drug interaction signatures for each cell line representing combinatorial data compiled in a 40 by 40 drug matrix. Upper right triangle, HMLE-shEcad cells, and lower left triangle, shGFP cells. Insets at left and right are magnified views of the nine concentration combinations for HMLE-shGFP and HMLE-shECad cells treated with AZD-7762 and Obatoclax. For each plot, nine horizontal bars plot the results of combining the two agents in all pairs of high (H), medium (M), and low (L) concentrations. The concentrations are listed in Supplementary Tables 1 and 2. Super- and sub-additivity is plotted using the Bliss independence model [11]. The yellow bar is the Bliss independent sum of growth inhibition for the two single agents measured in parallel; red indicates super-additivity, and green, antagonism. The blue A and O represent the rows in the table that correspond to AZD-7762 and obatoclax, respectively. (**C**) highest-ranked drug combinations. Links mark drug combinations with drug/dose observations surviving data filtering as described in the text and listed in Supplementary Table 3. The width of each link increases with the number of drug–dose pairs meeting filtering criteria. AZD-7762 and Tylophorine appeared in these combinations most frequently for HMLE-shEcad (pink) and HMLE-shGFP cells (light green), respectively, and are marked in red and green.

Since MSL cells are a majority population in CLBC, and phenotypic switching or selection of MSL cells may provide a drug-resistant phenotypic haven for BLBC, we have been interested in identifying agents that affect MSL cells preferentially or together with BL cells. In earlier work we reported IC_50_ values for growth inhibition of HMLE-shGFP control cells versus MSL HMLE-shEcad cells in three day CellTiterGlo ATP assays [9] (Supplementary Table 1 therein). We have now determined average area under the curve (AUC) in these experiments for percent inhibition plotted against log drug concentration (Figure 1A). AUC is a composite metric incorporating both potency and maximal effect, rather than potency alone. Generally, HMLE-shGFP control cells were more sensitive to growth inhibition than HMLE-shEcad cells with induced EMT. 24 agents out of 150 tested had comparable potency on both cell lines (IC_50_ under 5μM and within three-fold), suggesting that these agents, or others with the same target class, may be effective on TNBC cells in affecting both MSL cells and non-MSL cells. These include HSP90 inhibitor 17-AAG, IκB inhibitor Bay11-7085, proteasome inhibitor bortezomib, MEK inhibitor CIP 13-74, daunorubicin, doxorubicin, etoposide, FAK Inhibitor 14, eriocalyxin B, ixabepilone, methotrexate, mTorc inhibitors NV-128, NV-356, and NV360, obatoclax, paclitaxel, FGFR inhibitor PD173074, Stat3 inhibitor stattic, staurosporine, topotecan, triapine, trichostatin A, triptolide, tylophorine, vinblastine sulfate, and vorinostat (SAHA). It is noteworthy that among this group, doxorubicin, etoposide, ixabepilone, methotrexate, paclitaxel and vinblastine sulfate, are all US FDA-approved and most (the exception being etoposide) are standard-of-care agents for breast cancer in some settings. The impact of FDA-approved bortezomib, trichostatin A, vorinostat, and PD173074 suggests potential utility of proteasome inhibitors, HDAC inhibitors, and CDK inhibitors in TNBC. Three agents, JK184, eriocalyxin B, and AZD-7762 yielded lower IC_50_ for MSL HMLE-shEcad cells than for HMLE-shGFP cells. Noncanonical activation of GLI1 by NFκB [9] may explain sensitivity to JK184 and to eriocalyxin B, which has multiple reported targets including NFκB [10].

### Combinatorial screening

Since single agent therapy has had limited success for BL and CL breast cancer, we evaluated drug combinations. Based on the results of single agent screens, and aiming to maximize the diversity of drug targets, forty agents were chosen from the single agent set. They were evaluated by combinatorial screening in all pairwise combinations at three doses each, resulting in 7140 unique drug dose combinations (Figure 1B, Supplementary Table 1).

In Figure 1B, each drug combination is displayed with the nine drug-dose combinations (pairwise combinations of three doses). The Bliss sum of the single agent growth inhibition sum [11] determined in parallel is plotted yellow; super-additive results, red; and sub-additive effects, green. Overall, super-additive responses to drug combinations were less common with induced EMT in shECad (Supplementary Table 1), which is visually apparent from the reduced amount of red (super-additivity) and increased green (sub-additivity) in Figure 1B (compare upper right, HMLE-shEcad, to lower left, HMLE-shGFP). Combination data were filtered with the criteria that average growth inhibition for each single agent be between 15% and 75% (leaving space to observe super-additivity) and that the difference between theoretical Bliss sum [11] and observed GI is greater than 15%. Whereas for control HMLE-shGFP cells 19 agents at specific doses yielded super-additivity with five or more other agents, only eleven agents met this standard for HMLE-shEcad cells. Furthermore, the total number of times a particular drug-dose met the criteria was 186 for HMLE-shGFP cells but only 100 for HMLE-shEcad cells.

Biological differences between the two isogenic cell lines were evident, also, in the representation of individual agents at specific doses in combinations meeting filtering criteria (Supplementary Figure 1, Supplementary Tables 1–3). The most common combination partners for control HMLE-shGFP control cells were Tylophorine, mTOR inhibitor BGT226, FAK inhibitor 14, HMG CoA reductase inhibitor simvastatin, estrogen receptor antagonist tamoxifen, and pan-ERBB inhibitor lapatinib. In contrast, top-ranked HMLE-shEcad combination partners were checkpoint kinase inhibitor AZD-7762, diterpenoid, tyrosine kinase inhibitor bosutinib, pan-BCL2 inhibitor obatoclax, triapine, and STAT3 inhibitor Stattic.

Bioinformatic chord plot analysis linking drug sensitivities and cellular processes was next used to evaluate biological differences between shEcad and shGFP cells (Figure 1C). In this panel, top-ranked partner agents Tylophorine (shGFP) and AZD-7762 (shEcad) are emphasized with non-transparent green and red coloring, respectively. Differences in patterns of TKI sensitivity at least partly reflect differential expression of tyrosine kinases, including the EMT-induced reduction in ERBBs that we noted previously in shECad cells [9]. HMLE-shEcad cells were more sensitive to combinations including genotoxic agents (topotecan and mitomycin C), and to histone deacetylase inhibitor vorinostat, and Stat3 inhibitor Stattic. HMLE-shEcad cells are more sensitive to triptolide, triapine, and eriocalyxin B, each with multiple reported target processes. HMLE-shGFP were more commonly sensitive to the HMG CoA-reductase inhibitor simvastatin, which may operate through effects on synthesis of cholesterol and related metabolites, or through interference with isoprenylation of proteins including many important signaling proteins.

### Sensitivity of MSL cells to CHK1 inhibition

**Figure 2:**
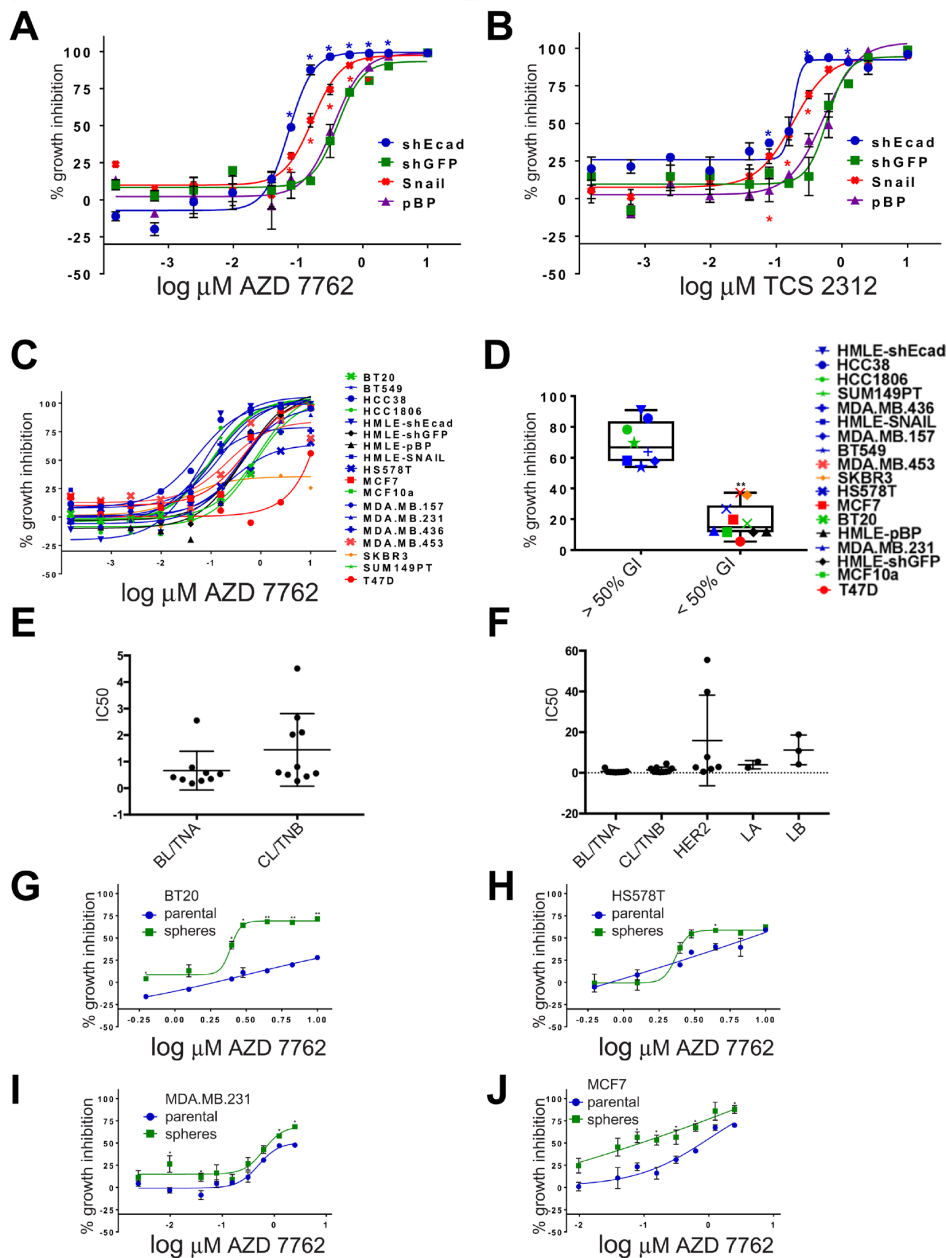
Dose-dependent sensitivity of breast cancer cell lines to CHK1 inhibitors. (**A**–**C**) dose-responses to AZD-7762 or TCS-2312 were determined on HMLE-shEcad, HMLE-shGFP, HMLE-Snail, HMLE-pBabePuro (pBP), and a series of human breast cell lines in three day CellTiter-Glo ATP assays [9] (^*^ *p <* 0.05, ^**^ *p <* 0.005). (**D**) growth inhibition of breast cancer cell lines incubated in 0.16 μM AZD-7762 in three day CellTiter-Glo assays. Black, HMLE control cells; blue, HMLE-induced EMT and CL cells; red, luminal; orange, HER2/ERBB2; green, basal-like (^**^ *p <* 0.005). (**E**, **F**) IC_50_ (μM) reported by Genomics of Drug Sensitivity in Cancer [13] for cell lines categorized by intrinsic transcriptional subtype [2, 34] as BL/TNA (HCC1187, HCC1143, HCC1937, HCC2157, HCC70, DU-4475, MFM-223, MDA-MB-468, BT-20), CL/TNB (CAL-120, HCC1395, BT-549, Hs-578-T, CAL-51, MDA-MB-361, HCC38, HDQ-P1, MDA-MB-231, CAL-86-1), HER2-enriched AU565, MDA-MB-453, HCC1569, HCC1954, HCC202, HCC2218, UACC-893), Luminal A (EFM-19, MDA-MB-175-V), or Luminal B (MDA-MB-330, EFM-192A, ZR-75-30) according to [2, 34]. GDSC cell lines were excluded if they were not described by subtype in [2, 34] or if the subtypes reported were discordant. Subtypes of some cell lines were only reported in one of the two publications. (**G**–**J**) Dose-responses to AZD-7762 of BT20, HS578T, MDA.MB.231 and MCF7 grown as adherent cells (parental, blue) or as non-adherent spheres (green) prior to plating in three day growth assays. Error bars represent standard error of the mean (^*^ *p <* 0.05, ^**^ *p <* 0.005).

Among all the agents tested, CHK1 inhibitor AZD-7762 formed the greatest number of super-additive drug combinations for HMLE-shEcad cells (Supplementary Table 1). As a single agent, this inhibitor was more potent on HMLE-shEcad cells (IC_50_ = 0.03 μM) versus HMLE-shGFP cells (IC_50_ = 0.09 μM) [9]. Since EMT induced with different genetic interventions results in related but distinct transcriptional phenotypes [12], we determined relative potency of AZD-7762 in a second set of HMLE cells with EMT induced by overexpression of SNAIL (Figure 2A). HMLE-Snail cells were more sensitive to growth inhibition by AZD-7762 than were pBabePuro control cells. To confirm that this is not an idiosyncratic characteristic of AZD-7762, we also tested a second CHK1 inhibitor, TCS-2312. This agent was also more potent on induced EMT lines HMLE-Snail and HMLE-shEcad than on parental controls (Figure 2B), supporting a general sensitivity of the induced-EMT lines to CHK1 inhibition.

### Sensitivity of TNBC cell lines to CHK1 inhibitors

With the overlap of CL and MSL phenotypes, we wondered whether TNBC cancer cell lines would also be sensitive to CHK1 inhibitors (Figure 2C, 2D). Eighteen breast cell lines were assayed for growth inhibition by AZD-7762 (Figure 2C, 2D). Generally, AZD-7762 was more potent on the induced EMT and CL cell lines than other breast lines, although HS578T and MDA.MB.231 were more resistant. Treatment of the lines with 0.16 μM AZD-7762 resulted in clustering of two distinct groups, those with GI50 values of greater or less than 50% (*p <* 0.05, *t*-test). EMT and CL lines were statistically over-represented in the grouping with GI50 values of greater than 50% compared to the group with GI50 values of less than 50% (Fisher’s exact test, *p <* 0.05) (Figure 2D). Basal-like lines HCC1806 and SUM149PT were relatively sensitive, with HS578T and (normal-like) MCF10a more resistant. Finally, AZD-7762 was less effective on luminal MDA.MB.453 cells, MCF7 cells, and was nearly ineffectual on luminal T47D cells and *HER2*-amplified SKBR3 cells.

AZD-7762 IC_50_ has been independently reported for an overlapping set of breast cancer cell lines in Genomics of Drug Sensitivity of Cancer (GDSC) [13]. Both BL and CL cell lines showed a bimodal distribution into more sensitive (IC_50_ < 1μM) and less sensitive (IC_50_ > 1μM) subsets (Figure 2E). The average IC_50_ for AZD-7762 was 0.66 μM (BL), 0.144 μM (CL), 3.98 μM (Luminal A/LA), 11.3 μM (Luminal B/LB) and 15.8 μM (HER2-E), (medians of B 0.41 μM; C: 0.5755 μM; H: 2.88 μM; LA 3.98 μM; LB: 10.8 μM) demonstrating a greater potency of this agent on BL and CL cells than other subtypes (Figure 2F).

Stem-like repopulating cells are thought to foster tumor resilience [8]. As induced EMT of HMLE cells concomitantly enriches for stem-like and tumor reconstituting characteristics [4], we hypothesize that AZD-7762 sensitivity is associated with acquisition of these characteristics. However, even though BT20, HS578T, and MDA.MB.231 cells are resistant to AZD-7762, mammospheres formed by these cells are growth suppressed by sub-μM concentrations of the drug (Figure 2G, 2H, 2I). Additionally, mammospheres grown from the luminal MCF7 cell line appear more sensitive to AZD-7762 compared to the parental/adherent line (Figure 2J).

### CHK1 inhibition selectively induces DNA damage in induced EMT cells

**Figure 3:**
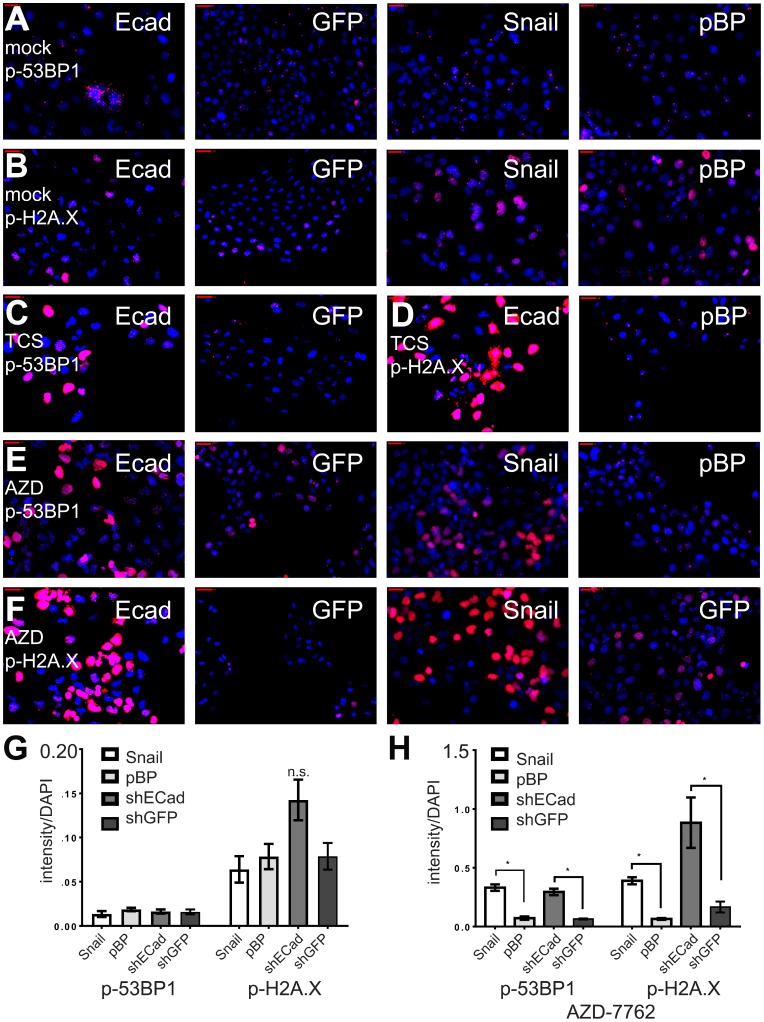
CHK1 inhibitors protect induced EMT cells from DNA damage. (**A**–**F**) Induced EMT and control HMLE cells were incubated with CHK1 inhibitors AZD-7762 (0.16 μM) or TCS-2312 (0.6 μM) for 24 hours, and analyzed by indirect immunofluorescence with anti-p-H2A.X or anti-p-53BP1 (red). Nuclei were stained with DAPI (blue). (**G**) intensities of p-53BP-1 or p-H2A.X relative to DAPI for untreated cell lines. (**H**) intensities of p-53BP1 and p-H2A.X after incubation for 24 hours with the IC_50_ dose for the HMLE-shEcad cells of AZD7762 = 0.16 μM. ^*^ *p <* .05 *t*-test, n.s. indicates not significant, *p* > 0.05.

**Figure 4:**
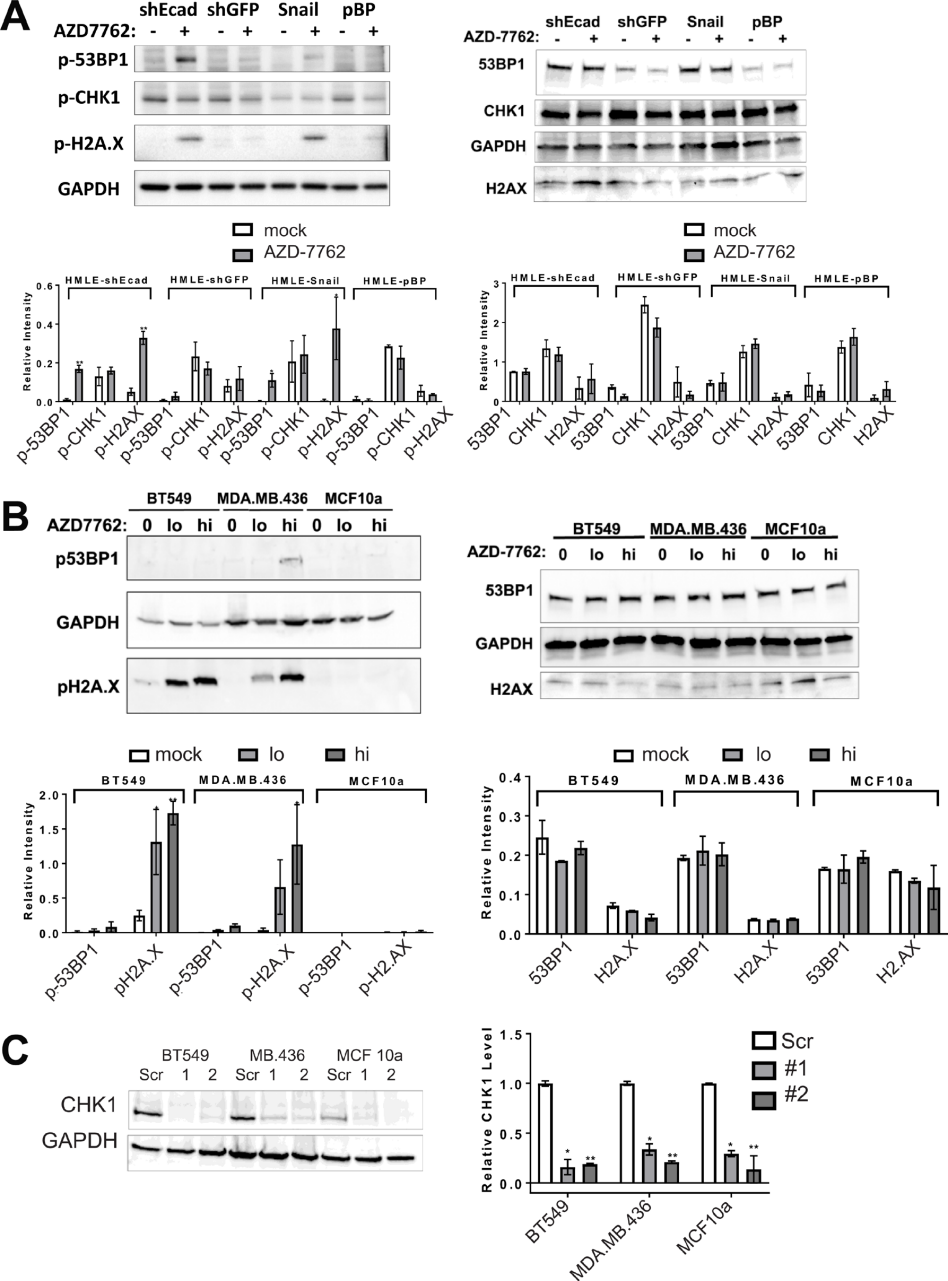
CHK1 inhibitors or *CHEK1* knockdown induce DNA damage in breast cancer cell lines. (**A**, **B**) immunoblots of lysates from induced EMT (HMLE-shEcad and HMLE-Snail) and control HMLE cells (HMLE-shGFP and HMLE-pBP), as well as CL (BT549, MDA.MB.436) and control (MCF10a) cell lines untreated or treated with 0.16 μM AZD-7762 for 16 h (**A**) or 0.08 (lo) or 0.16 μM (hi) AZD-7762 for 72 h (**B**). Antibodies were against p-53BP1, p-H2A.X, p-CHK1 or the unphosphorylated forms of these markers (53BP1, H2A.X, CHK1) as indicated, with GAPDH serving as a loading control. Quantification of immunoblots from at least three independent experiments is below each blot (^*^ *p <* 0.05, ^**^ *p <* 0.005). (**C)** CL cell lines BT549 and MDA.MB.436 and control MCF10a cells were infected with virus inducing knockdown of *CHEK1* (CHEK1sh1 and sh2) or with control scrambled virus. Degree of knockdown was quantified from three independent experiments (^*^ *p <* 0.05, ^**^ *p <* 0.005).

CHK1 signaling is activated in DNA damage, replicational stress, and mitotic spindle checkpoint pathways, leading to transient cell cycle arrest and DNA repair [14–16]. By interfering with cell cycle checkpoints, inhibition of CHK1 can promote premature passage through major cell cycle transitions leading to endogenous replicational stress and replication fork collapse and/or increased unrepaired DNA damage induced by exogenous sources. Moreover, TNBC are often characterized by defects in DNA repair owing to mutations in *TP53*, *BRCA1*, *BRCA2*, and other causes [17]. To determine if CL and MSL cells are intrinsically more sensitive to CHK1 inhibition due to heightened levels of endogenous DNA damage, we quantified the presence of phosphorylated 53BP1 (p-53BP1) and phosphorylated-H2A.X (p-H2A.X) in both immunofluorescence-based assays and in immunoblots. Induced EMT in HMLE-Snail or HMLE-shEcad cells did not significantly increase the frequency of DNA damage foci [18] (Figure 3A, 3B, 3G), nor did EMT induction heighten p-53BP1 or p-H2A.X levels, as detected by immunoblotting (Figure 4A). By contrast, formation of immunoreactive p-53BP1 and p-H2A.X foci in response to the CHK1 inhibitor TCS-2312 was greatly enhanced in induced EMT HMLE-shEcad cells relative to HMLE-shGFP cells (Figure 3C, 3D). Similarly, CHK1 inhibitor AZD-7762 preferentially induced p-53BP1 and p-H2A.X in induced EMT lines HMLE-shEcad and HMLE-Snail compared to control shGFP and HMLE-pBabePuro cells (Figure 3E, 3F, 3H). Additionally, AZD-7762 enhanced p-53BP1 and p-H2A.X as detected by immunoblotting in induced EMT HMLE-shEcad and HMLE-Snail cells, but not HMLE-shGFP or HMLE-pBabePuro cells (Figure 4A). Treatment with AZD-7762 did not alter unphosphorylated levels of these DNA damage markers in any of the HMLE lines to a significant extent (Figure 4A). AZD-7762 did not significantly lower the level of p-CHK1 in these assays, as treatment with higher doses of this drug resulted in dramatic cell death of the sensitive lines resulting in an ability to conduct an analysis of DNA damage markers. Despite this, the lower dose of the drug used here still resulted in dramatically increased levels of p-H2A.X and p-53BP1.

**Figure 5:**
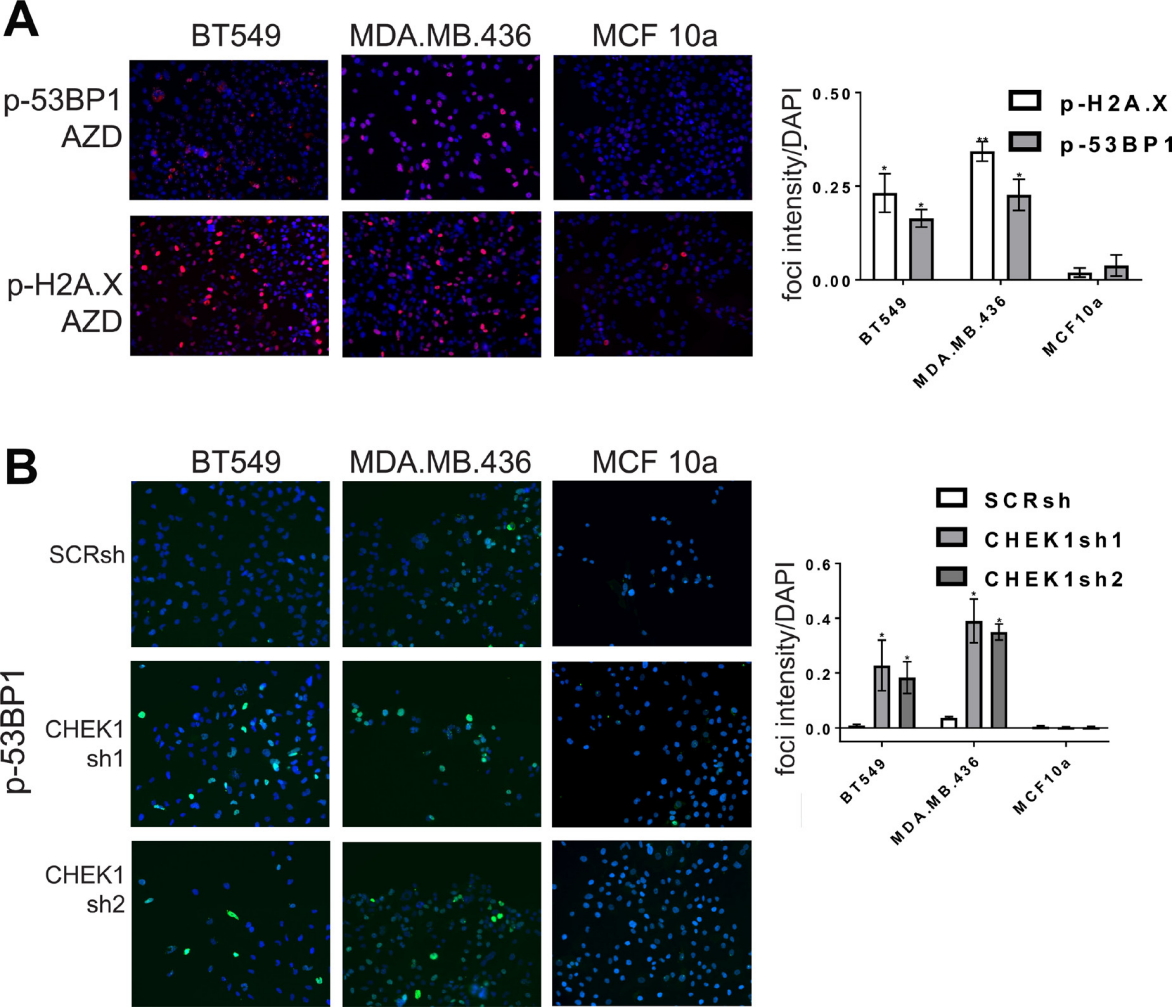
CHK1 inhibitors or *CHEK1* knockdown induce DNA damage in breast cancer cell lines. (**A**) CL cell lines BT549 and MDA.MB.436 and control MCF10a cells were incubated with AZD-7762 0.16 μM (**A**), or *CHEK1* knockdown was induced (**B**), for 72 hours and analyzed by indirect immunofluorescence with anti-p-H2A.X or anti-p-53BP1 (red, **A**) or anti-p-53BP1 (green, **B**). Nuclei were stained with DAPI (blue). The results from three independent experiments were quantified (^*^ *p <* 0.05, ^**^ *p <* 0.005).

We next determined whether AZD-7762 promotes DNA damage in TNBC cell lines sensitive to the drug. AZD-7762 induced a dose-dependent accumulation of p-H2A.X detected by immunoblotting in BT549 and MDA-MB-436 cells, and p-53BP1 in MDA.MB.436 cells, while not significantly altering unphosphorylated levels of these markers (Figure 4B). AZD-7762 induced phosphorylation of H2A.X and 53BP1, and promoted p-53BP1 and p-H2A.X focus formation in sensitive CL BT549 and MDA.MB.436 cells (Figure 5A), but not resistant (non-transformed) MCF10a cells (Figure 5A). Finally, knockdown of *CHEK1*, but not a scrambled shRNA control, induced immunoreactive p-H2A.X in BT549 cells and MDA.MB.436 cells (Figure 4C and Figure 5B), similar to AZD-7762 treatments. Overall, these results show that CL and induced EMT breast cells exhibit greater sensitivity to CHK1 inhibitors relative to normal mammary cells, and that this is associated with the acquisition of DNA damage, likely from on-target inhibition of CHK1 by these agents.

### Impact of checkpoint kinase inhibitors on cell cycle

**Figure 6:**
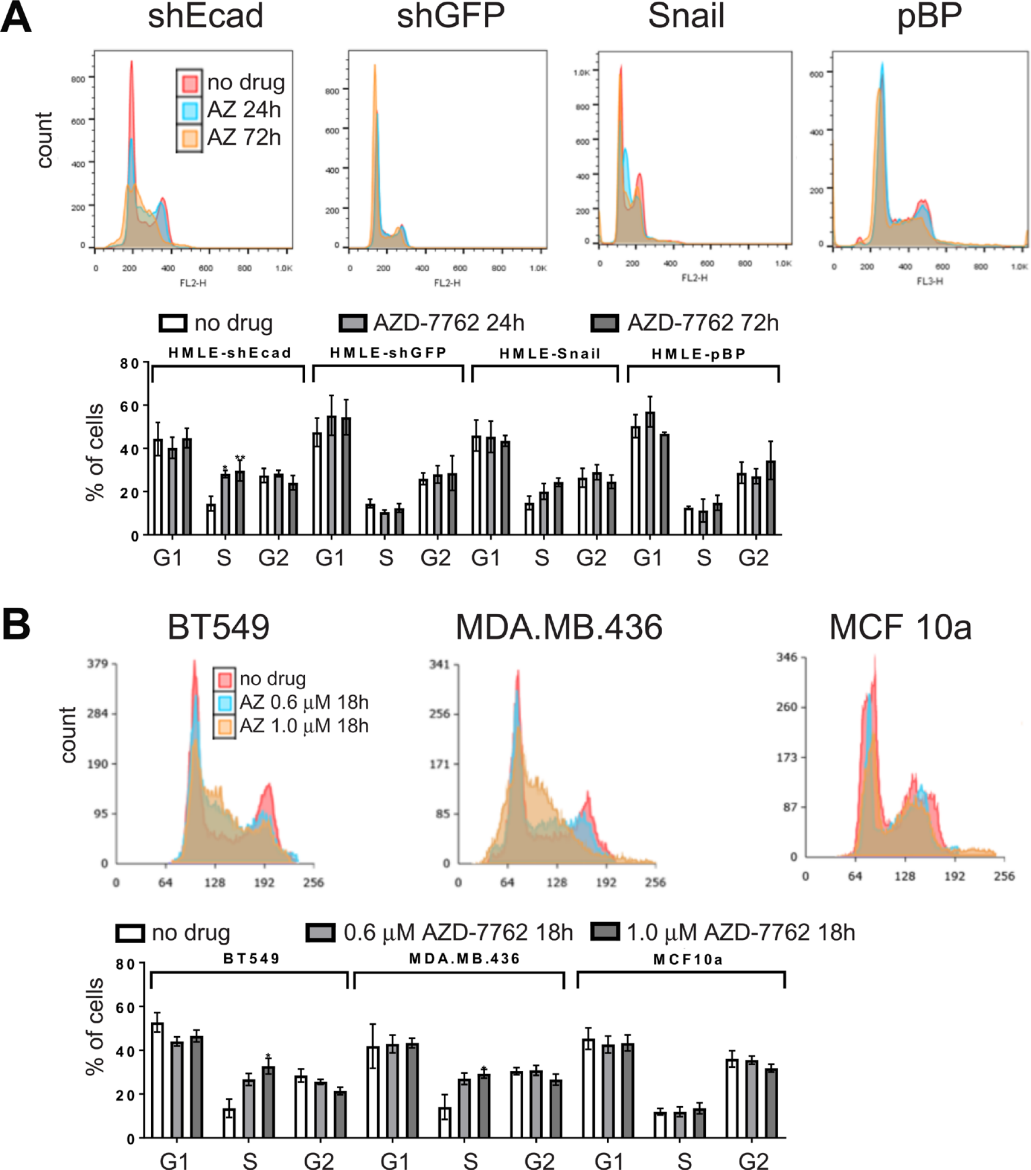
Cell cycle analysis. DNA content/cell-cycle analysis of induced EMT (HMLE-shEcad and HMLE-Snail) and control HMLE cells (HMLE-shGFP and HMLE-pBP) (**A**), as well as CL (BT549, MDA.MB.436) and control (MCF10a) cell lines (**B**) treated with 0.16 μM AZD-7762 for 0, 24, or 72 hours (A), or with no drug, 0.6 μM or 1.0 μM AZD-7762 for 18 hours (B). Histogram overlays were created in Adobe Photoshop. The results from three independent experiments were quantified (^*^ *p <* 0.05, ^**^ *p <* 0.005).

CHK1 is activated in response to DNA damage, replicational stress, and the spindle checkpoint to mediate protective cell cycle arrest [14–16]. Checkpoint failure can lead to increased DNA damage from unrepaired DNA damage or replication fork collapse, and chromosomal damage from premature mitotic entry [19]. We determined whether the greater sensitivity of HMLE-shEcad and HMLE-Snail cells to AZD-7762, is associated with cell cycle alterations. Treatment of HMLE-shEcad or HMLE-Snail cells with AZD-7762 for 24 hours resulted in accumulation of cells with S-phase DNA content that was further enhanced over 72 hours (Figure 6A). These results were more dramatic on the HMLE-shEcad cells as the dosing used was for the IC50 value for HMLE-shEcad cells, while the HMLE-Snail cells are slightly less sensitive to AZD-7762 (Figure 2A). Similarly, CL BT549 and MDA.MB.436 cells showed a proportionate increase in S-phase DNA content compared to AZD-7762-resistant and non-transformed MCF10a cells (Figure 6B). We conclude that AZD-7762 sensitivity, characteristic of induced EMT and CL cells, is at least partly a consequence of premature S-phase entry or cell cycle arrest within S-phase associated with defective CHK1 regulation.

### Drug combinations with CHK1 inhibitor

As dose-limiting toxicities have been an issue for AZD-7762 and for other CHK1 inhibitors in early phases of human clinical testing [20, 21], we sought partner agents that would enhance utility of CHK1 inhibitors. In our combinatorial screen, combination partners with AZD-7762 yielding the greatest impact for HMLE-shEcad cells included the anti-apoptotic BCL-2 inhibitor obatoclax/GX15-070, src family tyrosine kinase inhibitor bosutinib, FAK inhibitor 14, Triptolide, STAT3 inhibitor Stattic, topoisomerase inhibitor topotecan, mitomycin C, broad CDK inhibitor flavopiridol, and a diterpenoid (Supplementary Table 1). We decided to evaluate the AZD-7762/BCL-2 inhibitor combination further, as the US FDA has approved BCL-2 inhibitor venetoclax/ABT 199 for subsets of lymphocytic leukemia, and clinical trials are underway to evaluate CHK1 inhibitors.

**Figure 7:**
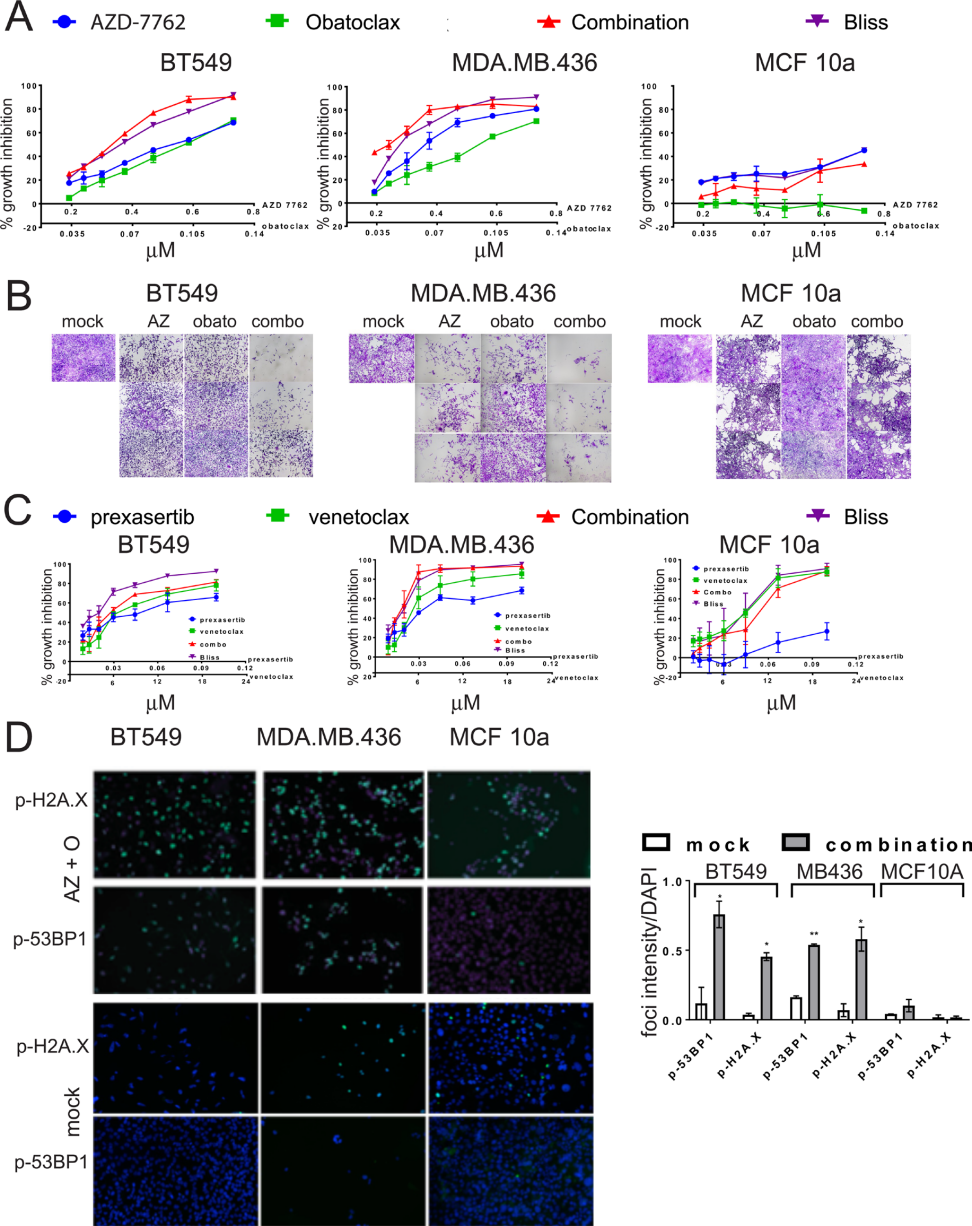
Growth inhibition of TNBC cell lines by combinations of CHK1 inhibitors and BCL-2 inhibitors. (**A**) Dose-responses of CL (BT549, MDA.MB.436) and control (MCF10a) cell lines exposed to AZD-7762, obatoclax, or a combination of both agents. Bliss additivity for each dose combination was calculated from the single-agent responses. Error bars represent standard error of the mean. (**B**) photomicrographs of crystal-violet stained wells from (**A**), with the highest three doses imaged. (**C**) Dose-responses of CL (BT549, MDA.MB.436) and control (MCF10a) cell lines exposed to prexasertib, venetoclax, or a combination of both agents. Bliss additivity for each dose combination was calculated from the single-agent responses. Error bars represent standard error of the mean. (**D**) CL cell lines BT549 and MDA.MB.436 and control MCF10a cells were incubated with 0.3 μM AZD7762 and 0.05 μM obatoclax for 72 hours, and analyzed by indirect immunofluorescence with anti-p-H2A.X or anti-p-53BP1 (green). Nuclei were stained with DAPI (blue). Foci intensity relative to DAPI was quantified (^*^ *p <* 0.05, ^**^ *p <* 0.005).

**Figure 8:**
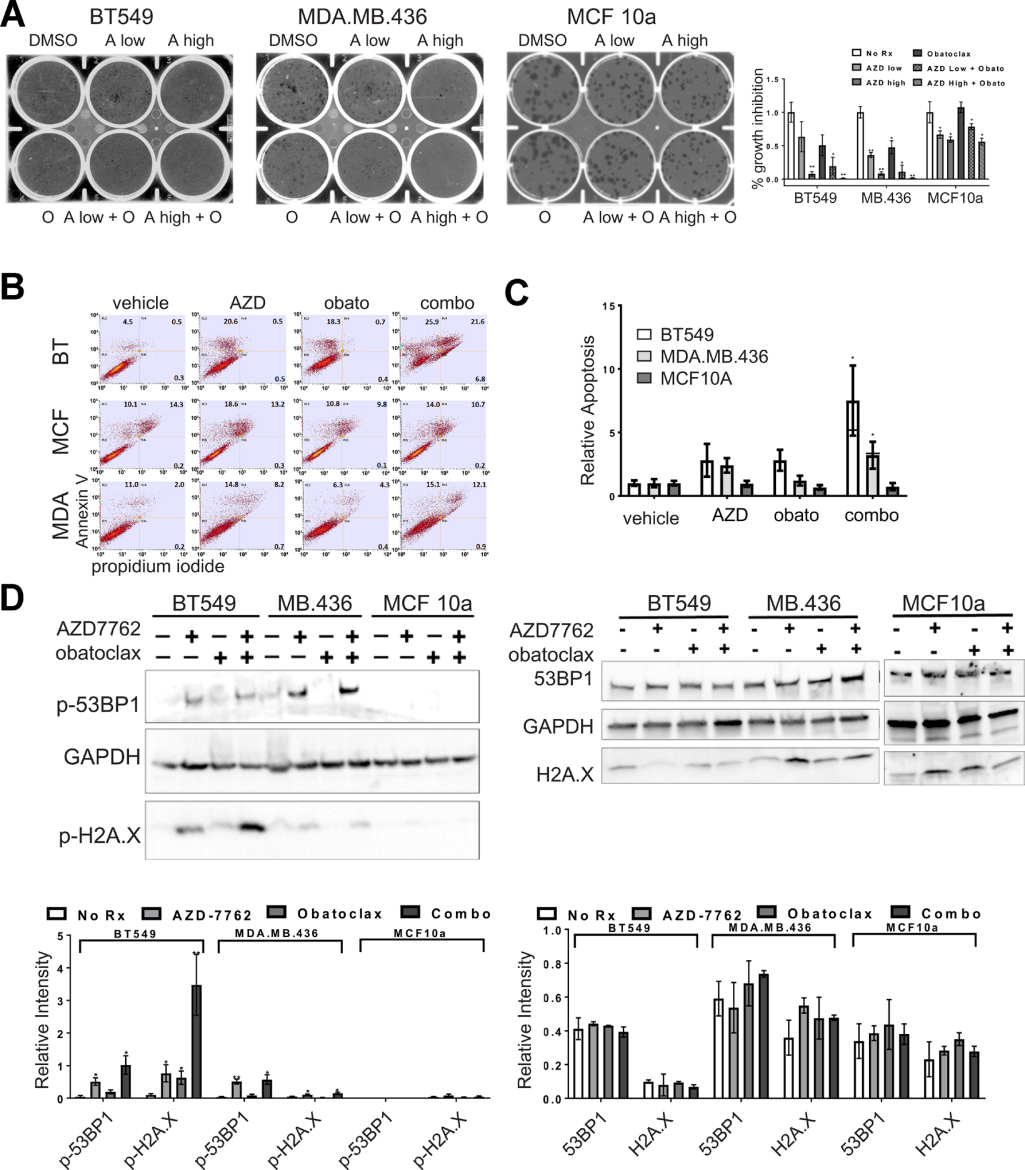
Single agent and combination treatment with AZD-7762 and obatoclax. (**A**) Clonogenicity assays of CL (BT549, MDA.MB.436) and control (MCF10a) cell lines exposed to low (0.15 μM) or high (0.3 μM) doses of AZD-7762, 0.025 μM obatoclax, or a combination of both agents. Limiting dilutions of cells were plated and colonies were allowed to form for 10 days prior to staining with crystal violet and imaging. The results from three independent experiments were quantified (^*^ *p* < 0.05, ^**^ *p* < 0.005). (**B**) Apoptosis analysis of CL (BT549, MDA.MB.436) and control (MCF10a) cells stained with propidium iodide and FITC-Annexin-V. Cells were treated for seven days with 0.3 μM AZD7762, 0.05 μM obatoclax or with both agents. (**C**) Quantification of all three quadrants (PI+, AV+, and both PI and AV+) from (**B**). Averages are plotted relative to vehicle control. The bar for the combination treatment indicates level expected from Bliss additivity of the single agent treatments. Error bars represent standard error of the mean (^*^ *p* < 0.05). (**D**) Immunoblot of CL (BT549, MDA.MB.436) and control (MCF10a) cell lysates treated for 72 hours with 0.3 μM AZD7762, 0.05 μM obatoclax, or both agents. Antibodies were against p-53BP1, and p-H2A.X, or the unphosphorylated forms of these markers (53BP1, H2A.X) as indicated, with GAPDH serving as a loading control. Quantification of immunoblots from at least three independent experiments is below each blot (^*^ *p* < 0.05, ^**^ *p* < 0.005).

The combination of AZD-7762 plus obatoclax inhibited growth of sensitive BT549 and MDA.MB.436 cells, but had only moderate impact on MCF10a cells, even at high concentrations (Figure 7A, 7B). Similarly, combination of a second CHK1 inhibitor, prexasertib, currently in clinical trials, with venetoclax (or with obatoclax) confirms that multiple agents from the same target class combine more effectively (Figure 7C). Both the combinations of AZD-7762 with obatoclax and prexasertib with venetoclax resulted in several combination index (CI) values indicative of synergy at effective growth inhibitory levels (Supplementary Table 4). The AZD-7762 combination super-additively suppressed clonogenic growth of BT549 and MDA.MB.436 cells, while MCF10a cells were relatively unaffected (Figure 8A). The relative sensitivity of CL lines MDA.MB.436 and BT549 to AZD-7762 was reflected in preferential apoptosis of these cell lines in comparison to normal-like MCF10a cells, which have basal characteristics (Figure 8B, 8C). While cell death can be achieved by treatment with AZD-7762 alone, the combination of AZD-7762 plus obatoclax resulted in super-additive effects in terms of apoptosis (Figure 8B, 8C). The combination also appears to increase DNA damage, as p-53BP1 and p-H2A.X levels and foci are increased in sensitive BT549 and MDA.MB.436 cells, but not resistant MCF10a cells (Figures 7D, 8D).

## DISCUSSION

TNBC poses one of the foremost breast cancer treatment challenges. The most common subsets, BLBC and CL/MSL BC, comprise approximately 75% of TNBC. MSL cells may be important both as the majority population in CLBC and as a minority subpopulation that may disproportionately contribute to tumor reconstitution after treatment and may act as metastatic pioneers. Building on an earlier screen of 150 single agents with a pair-wise combinatorial screen of 40 agents at multiple concentrations, we identified drug combinations that preferentially affect HMLE-shEcad cells with EMT induced by E-cadherin knockdown. HMLE-shEcad cells were more sensitive to drug combinations including genotoxic agents, a histone deacetylase inhibitor, and a STAT3 inhibitor.

We focused on CHK1 inhibitors as they were more potent for induced EMT HMLE-shEcad and HMLE-Snail cells than for control cells; they were top-ranked as combining agents for HMLE-shEcad; and because they are currently under investigation in clinical trials. Induced EMT lines were remarkably more sensitive to single agent CHK1 inhibitors in monolayer growth, clonogenicity, and apoptosis assays compared to isogenic control cells. Growth inhibition of induced EMT HMLE cells and sensitive breast cancer cell lines by CHK1 inhibitors was associated with formation of p-H2A.X and p-53BP1 DNA damage response foci and pan-nuclear staining, and with enrichment for cells in S-phase. Crucially, these phenotypes are observed upon CHK1 inhibition in the absence of exogenous DNA damaging agents. CHK1 is phosphorylated by ATR in response to DNA damage. However, CHK1 also plays an essential role in the absence of DNA damage, whereby it limits oncogene-induced replication stress by maintaining replication forks and facilitating restart of stalled replication forks. As we did not observe a difference in baseline levels of p-H2A.X and p-53BP1 between induced EMT HMLE cells and control HMLE cells, and there was no observable cell-cycle difference between these lines, the effectiveness of CHK1 inhibition as a single agent on induced EMT lines is likely due to the role of CHK1 in maintenance of replication fork progression rather than the DNA damage response. The pan-nuclear staining of p-H2A.X and p-53BP1 in induced EMT HMLE cells in response to CHK1 inhibition is indicative of replication stress, not of dsDNA breaks, as several groups have shown that such staining is independent of dsDNA breaks and/or only occurs in S-phase cells [22–24]. This suggests that CHK1 is needed in MSL cells to allow proper fork progression, and in the absence of CHK1 function replication stress and ultimately cell death results.

Cellular responses to CHK1 inhibition are influenced by mutations in DNA damage response genes, and *TP53* mutation has been implicated in sensitivity to CHK1 inhibition in TNBC [25]. Approximately 80% of TNBCs harbor inactivating *TP53* mutations and are defective in double-strand DNA break repair. *TP53* status does not however explain the differential sensitivity of EMT-induced HMLE cell lines to specific single agents or to drug combinations including CHK1 inhibitors, since *both* HMLE and induced EMT lines express SV40 large T antigen (LT), which inactivates RB1 and p53, and small t (t) antigens.

Since MSL cells predominate in CLBC and constitute a minority population in BLBC, we determined whether tumor cell lines classified as CL/TNB or BL/TNA are sensitive to CHK1 inhibitors. In our hands, and in data reported through GDSC, BL and CL cells tend to be more sensitive to AZD-7762 than HER2, luminal A, and luminal B cells and the majority of BL and CL cell lines responded to sub-μM concentrations of AZD-7762. Exact values between our data and GDSC data are somewhat inconsistent, likely due to differences in experimental and/or analytical procedures, but the data agree well in ordering sensitivity of cell lines to AZD-7762. Both our data and the GDSC dataset show that most CL cell lines are sensitive to CHK1 inhibition, while the CL line MDA.MB.231 is relatively resistant. Additionally both our data and GDSC data identify the luminal T47D line as being remarkably insensitive to AZD-7762.

A minor subset of CL and BL lines including HS578T and BT20 cells were somewhat resistant to CHK1 inhibition in three day growth experiments. Nonetheless, when non-adherent mammospheres, which are rich in tumor-reconstituting cells, were grown from these lines, the sphere cells displayed significantly greater sensitivity to AZD-7762 (at slightly longer exposures) than was observed in parental lines. Additionally spheres grown from the rather resistant luminal MCF7 cell line were somewhat more sensitive than the adherent parental line. Mammospheres are enriched for CL/MSL transcriptional phenotype [1]. Hence CHK1 inhibitors may be useful both for CL/MSL tumors, but also for other TNBC for which CL/MSL cells are a minority population but important for tumor repopulation post-treatment. Indeed, our results are well-aligned with a recent separate independent study that determined CHK1 and the DNA repair pathway as the most commonly deregulated pathway in TNBC [26].

We find that induced EMT cells and CL lines are sensitized to cell cycle dysregulation that amplifies DNA damage and replication stress induced by CHK1 inhibition, suggesting that CHK1 inhibition could be a therapeutic target for patients with CLBC or TNBC. Indeed, several clinical trials are underway examining the efficacy of CHK1 inhibitors, despite initial failings of first-generation CHK1 inhibitors due to dose-limiting toxicities. A strategy towards best leveraging CHK1 inhibitors in the future may be in combining them with partner agents. Additionally, since resistance to targeted therapy is an enduring problem in the clinic, combination treatments may be effective in reducing incidence of resistance. We therefore wanted to explore agents that could effectively combine with CHK1 inhibition. AZD-7762 has multiple effective partners, including with agents within the cell-cycle category as well as connections to agents in the DNA/RNA category (Figure 1C). The combination of AZD-7762 with the pro-apoptotic agent obatoclax was the most prominent (Figure 1C), indicating many effective and super-additive combinatorial doses. We provide evidence that a CHK1 inhibitor in addition to an anti-BCL2/pro-apoptotic agent has super-additive and/or synergistic effects, as evidenced by the combination of AZD-7762 and obatoclax outperforming Bliss additivity in terms of growth inhibition, colony formation, and apoptosis assays (Figures 7, 8). Obatoclax on its own was not selective nor particularly effective at the doses used. Importantly, these data were not restricted to AZD-7762 and obatoclax, as prexasertib (currently in clinical trials) and FDA-approved venetoclax showed super-additive effects as well.

Most prior work on CHK1 inhibitors combines them with a DNA-damaging agent such as gemcitabine. The idea being that cells with damage will bypass the S and G2-checkpoints when CHK1 activity is inhibited, prematurely enter mitosis, and experience mitotic catastrophe and ultimately cell death. Our data indicate however that CHK1 inhibitors are effective as single agents, without the addition of a DNA damaging agent. Although we did identify some partnering agents that induce DNA damage, such as the DNA crosslinker mitomycin C and the topoisomerase inhibitor topotecan, the majority of the top partnering agents do not induce DNA damage. These latter regimens might be better tolerated, as CHK1 inhibition in combination with gemicitabine led to a higher frequency of adverse effects than expected for gemcitabine treatment alone [27]. For example, we identified the src inhibitor bosutinib and the Stat3 inhibitor stattic as top partnering agents with AZD-7762. Additionally, partnering with a non-DNA damaging agent could be beneficial, as we have some evidence that the combination of a DNA damaging agent plus AZD-7762 removes the observed selectivity for induced EMT cells, such as the combination of AZD-7762 and mitomycin C, or daunorubicin. Selectivity was maintained when we treated with a CHK1 inhibitor plus a pro-apoptotic agent, indicating that this treatment regimen may specifically target MSL cells while leaving non-tumorigenic cells unharmed.

There is no consensus on best selection criteria for CHK1 inhibitors (reviewed in [28]). Based on preclinical observations, susceptibility to these inhibitors may be associated with loss of G1/S checkpoints, for example conferred by TP53 and RB mutations, or aberrant G2/M regulation or checkpoint defects. In the context of our findings, susceptibility may also be associated with a significant MSL subpopulation.

Decades after the importance of CSC and EMT were originally proposed, the original models remain as important conceptual guides, but they are oversimplified [8]. The heterogeneity of clonal tumor cell populations is now well-established, and lineage tracing experiments have revealed the important contribution of subclonal lineages in establishing bulk tumor populations and in restoring these populations after treatment. The founders of tumor reconstituting clones often have characteristics of endogenous stem cells responsible for normal tissue maintenance. A finer view, however, has revealed unexpected cellular plasticity: cellular differentiation hierarchies are not so rigidly structured and vertically organized as originally believed. CSCs can be derived from normal-like stem cells, but in other cases from de-differentiation or trans-differentiation from other related cell types. Single cell profiling technologies are revealing an extraordinary diversity of tumor cells and gross phenotypic states. For example, mesenchymally-transformed tumor cells encompass cells with a range of epithelial, metastatic, and tissue reconstituting abilities [29]. Cell lines in which EMT has been induced with different transcription factors differ phenotypically, and different EMT-inducing transcription factors make different contributions to tumorigenesis [30].

While the plasticity of tumor cells poses great challenges to managing TNBC and other cancers, the preferential drug sensitivities of minority or transitory tumor subpopulations reveal new sets of drug targets, provided that they are obligatory or at least common sources of tumor resilience post-treatment, or are important intermediaries in tumor progression. The drug combinations we have identified by screening on MSL breast cancer that are also active on TNBC cell lines offer new opportunities for control of TNBC.

EMT cells and CSC are generally associated with drug resistance, with mechanisms including greater drug efflux and slower cell cycling than more differentiated tumor cells. An EMT core signature derived from induced EMT HMLE cells is associated with treatment resistance [12]. This report describes differential and common drug sensitivities of paired cell lines modeling EMT. We have identified a number of drug combinations that selectively suppress growth of control cells and MSL cells with induced EMT. A combination of checkpoint kinase inhibition with pro-apoptotic agents preferentially affected MSL cells and CL cells, and present a plausible combination for managing many triple-negative breast cancers.

## MATERIALS AND METHODS

### Cell culture

HMLE-shEcad, HMLE-shGFP, HMLE-Snail, and HMLE-pBabe-Puro cell lines were a gift from Robert A. Weinberg (Massachusetts Institute of Technology, Cambridge, MA) and were propagated as previously described [3, 4]. SUM149PT cells were obtained from BIOIVT. All other cell lines were obtained from American Type Culture Collection and propagated according to instructions. All experiments were conducted on low-passage cells.

### Single agent screen

Single agent screening has been described [9]. For AUC calculations, parameter log- logistic curves were fit with the R drc library. AUC was calculated with numeric integration, using the base R package, from the EC_10_ to the highest concentration used. The model-free AUC method was used, which is the sum of the inhibition values divided by the number of values. These “AUC average” values were used for further analyses. The plot in Figure 1C was produced using R circlize [31] and R ColorBrewer packages. This diagram includes agent/dose combinations meeting filtering criteria of average growth inhibition for each single agent between 15% and 80% and measured growth inhibition at least 10% greater than calculated Bliss summed growth inhibition, slightly less stringent than for Supplementary Figure 1.

### Combinatorial screen

Combinatorial drug screening was performed at the Yale Center for Molecular Discovery (West Haven, CT) as previously described [32]. Briefly, robotic hit-picking was used to set up twenty-two master 384-well master plates for all combinations of forty agents at three doses each including single agents at the same concentrations, negative controls (0.1% DMSO) and positive controls for maximal cell killing (20% DMSO). Twenty-two master 384-well screening plates were set up with these combinations. HMLE-shEcad and HMLE-shGFP cell lines were plated in triplicate at a density of 750 cells per well in 384 well plates. Drug combinations were pin-transferred from master plates into 384-well plates with shGFP or shEcad cells for CellTiterGlo assays, performed in triplicate as described before [32]. Three days after drug addition, cell accumulation/viability was assayed using CellTiter-Glo reagent (Promega), which measures ATP. Only experiments with high Z-factor quality indices (>0.5) were analyzed. Data were analyzed as described previously [32].

### Manual growth inhibition assays

Cells were seeded in clear-bottomed 96-well plates at a density of 1,000 cells/well. Agents were added the following day. Three days later, cells were assayed by CellTiter-Glo reagent as previously described [9] or via crystal violet staining. Briefly, following two washes with phosphate buffered saline (PBS), cells were fixed in ice-cold methanol for 10 minutes. Cells were then stained in 0.5% crystal violet for 10 min. Excess stain was removed by repeated washings in water. Plates were air-dried prior to allow solubilization of stain by addition of a 10% acetic acid solution. Absorbance was measured at 590 nm. Dose-response curves were generated using GraphPad Prism. Identification of drug synergy was assessed using the Bliss independence model [11], or the Chou-Talalay method [33] using normalized isobologram analyses.

### Immunofluorescence and immunoblotting

Immunofluorescence assays were performed as described previously [9]. Primary antibodies against p-H2A.X (Ser139), and p-53BP1 (Ser1778) (Cell Signaling Technology), were diluted 1:250 with PBS with 0.1% Tween-20 (PBST), and incubated overnight at 4° C. Secondary antibodies were either Alexa Fluor 594-conjugated secondary (Invitrogen), DyLight^TM^ 488 (Rockland) or DyLight™ 549, diluted 1:1000 in PBST. Slides were mounted with Prolong Gold (Invitrogen). Cellular lysates were produced and analyzed by immunoblotting as described previously [9]. Primary antibodies were against p-H2A.X (Ser139), H2A.X, p-53BP1 (Ser1778), 53BP1, CHK1 (Cell Signaling Technology) p-CHK1 (Ser345, R&D Systems) and GAPDH (Santa Cruz). Results were quantified using ImageJ and Image Lab from BioRad.

### Apoptosis analysis

Apoptosis was analyzed as previously described [9]. Cells were plated at 5 × 10^4^ cells/well in 6-well format and allowed to adhere overnight. The following day cells were treated for the doses and times indicated.

### Cell cycle analysis

Following treatment, cells were harvested by trypsinization, washed in 1x PBS, and resuspended in ice cold 1x PBS. To this, ice cold ethanol was added while vortexing. Cells were stored at –20° C until analysis. Fixed cells were spun down, washed in 1x PBS, and resuspended in 100 μg/mL RNase A, 0.1% Triton X-100, 40 μg/mL propidium iodide solution in 1x PBS for 30’ at room temperature (RT). After incubation in the staining solution, 100 μg/mL RNase A, 0.1% Triton X-100 solution was added and cells were analyzed on a BioRad S3e Cell Sorter for DNA content analysis. For each treatment, 15,000 cells were analyzed. The proportion of cells in each stage of the cell cycle (1n, 1-2n, and >2n) was determined using FloJo and ProSort software from BioRad.

### Clonogenicity assay

Colony forming ability was assayed as previously described [9]. Briefly, limiting dilutions of cells were plated in 6-well plates, and the following day inhibitory agents were added. Colonies were allowed to grow for 10-12 days, then were stained with crystal violet and imaged. Results were quantified using ImageJ.

### *CHEK1* knockdown

For CHEK1 knockdown, the RHS4430-200209571 - V2LHS_112996 and RHS4430-200276944 - V3LHS_339765 GIPZ vectors were purchased from Thermo Fisher Scientific and cloned into pInducer10 via MluI and XhoI. The resulting plasmids were co-transfected with viral packaging plasmids into 293T cells and virus was harvested as previously described [9]. Cells were infected, selected, and knockdown was induced as previously described [9]. Knockdown was quantified using Image Ready by BioRad.

### Sphere assays

Mammospheres were grown under non-adherent conditions as previously described [9]. Spheres were harvested following manual agitation using a pipette tip, and then collected with a 35 micron mesh filter. Spheres were dissociated with trypsin to a single-cell suspension, and then plated in 96-well plates for cell proliferation analysis as described above.

## SUPPLEMENTARY MATERIALS











## Abbreviations

53BP1: p53 binding protein 1
AUC: area under the curve
BCL2: B cell lymphoma 2
BL: basal-like
BLBC: basal-like breast cancer
CHK1: Checkpoint kinase 1
CL: claudin-low
CLBC: claudin-low breast cancer
DMSO: dimethyl sulfoxide
EGFR: epidermal growth factor receptor
EMT: epithelial to mesenchymal transition
ER: estrogen receptor
ERBB2: epidermal growth factor receptor 2
FAK: focal adhesion kinase
GLI1: glioma associated oncogene 1
H2A.X: histone H2A.X
HER2: human epidermal growth factor receptor 2
HMLE: human mammary epithelial cells transformed with large T and small t antigens
MSL: Mesenchymal stem-like
NFκB: nuclear factor kappa-light-chain-enhancer of activated B cells
PBS(T): phosphate buffered saline (with 0.1% Tween-20)
PR: progesterone receptor
RT: room temperature
sh: short hairpin
TKI: tyrosine kinase inhibitors
TNBC: triple-negative breast cancer
