# Supplementary material for: Combinatorial drug screening of mammary cells with induced mesenchymal transformation to identify drug combinations for triple-negative breast cancer
Source: Oncotarget. 2019 Aug 6;10(47):4822–39. doi: 10.18632/oncotarget.27104 (PMC6690678; doi:10.18632/oncotarget.27104)
Supplement: Supplementary file 1 [file oncotarget-10-4822-s001.pdf]

Combinatorial drug screening of mammary cells with induced mesenchymal transformation to identify drug combinations for triple-negative breast cancer

SUPPLEMENTARY MATERIALS

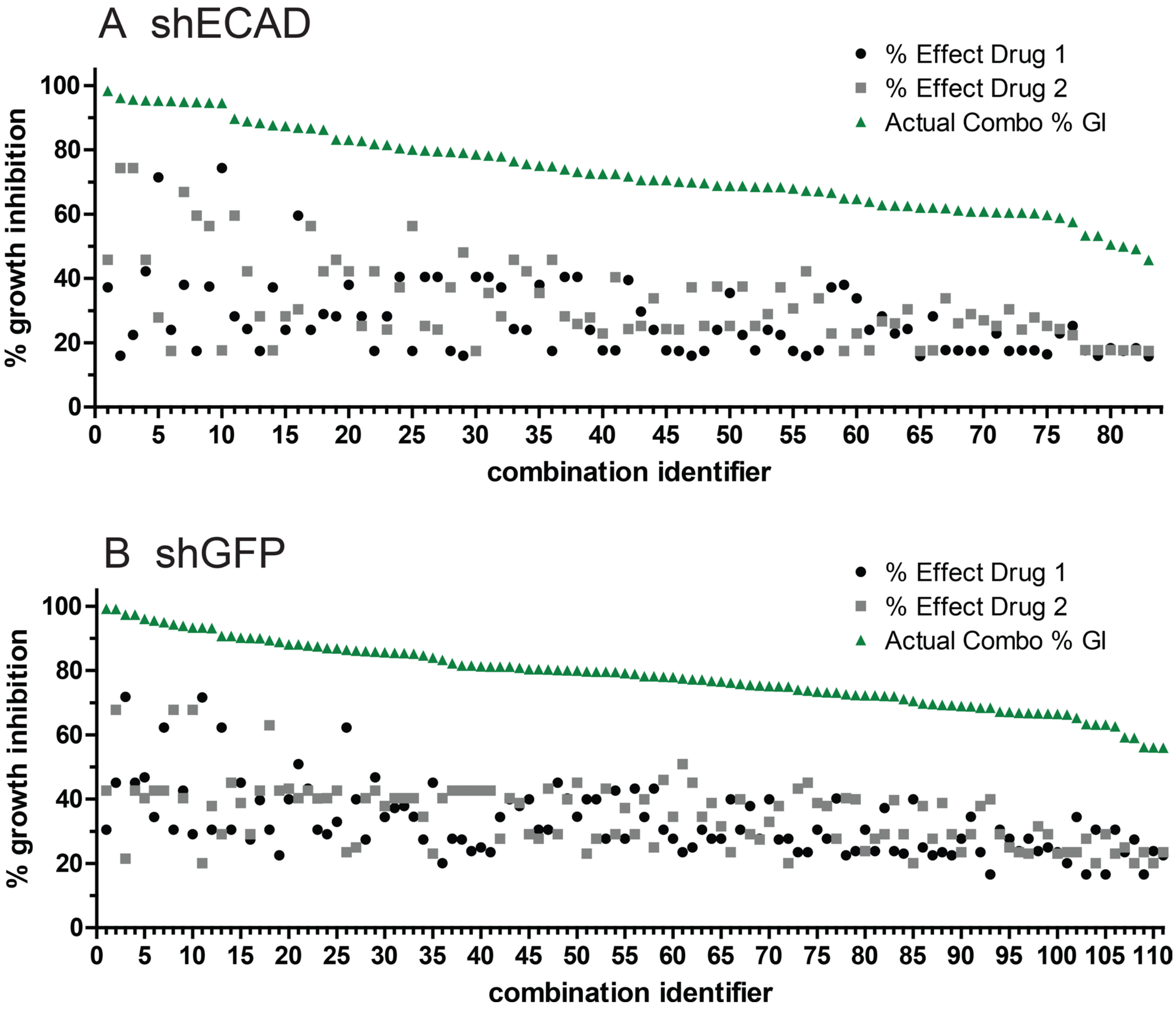

Supplementary Figure 1: Single agent and combination growth inhibition for individual drug-dose combinations. (PDF) Individual drug-dose combinations that meet filtering criteria described in the text are ranked in order of decreasing percent growth inhibition for HMLE-shECad cells (**A**) and HMLE-SHGFP cells (**B**). "Combination identifier" numbers refer to the Combination ID columns in Table S1 for HMLE-shECad cells and Additional File 3: Table S2 for HMLE-shGFP cells, which list the specific agents and doses. Percent growth inhibition is measured for single agents (Agent 1, black circles; agent 2, grey squares; combination, green triangles).

Supplementary Table 1: Drug combinations HMLE-shECad cells. (XLSX 20 kb) Drug dose combinations, with drug concentrations that met filtering criteria described in the text. "Low", "Med", "High" describe whether these were the lowest, intermediate, or highest concentration tested for the agent. "Combination ID" numbers correspond to "combination identifier" numbers in Supplementary Figure 1A. "% Effect" of drug combination or each single agent measured in parallel at the same dose is calculated relative to 100% maximal growth inhibition for positive control. See Supplementary Table 1

Supplementary Table 2: Drug combinations HMLE-shGFP cells. (XLSX 22 kb) Drug dose combinations, with drug concentrations that met filtering criteria described in the text. "Low", "Med", "High" describe whether these were the lowest, intermediate, or highest concentration tested for the agent. "Combination ID" numbers correspond to "combination identifier" numbers in Supplementary Figure 1B. "% Effect" of drug combination or each single agent measured in parallel at the same dose is calculated relative to 100% maximal growth inhibition for positive control. See Supplementary Table 2

Supplementary Table 3: Individual agents at specific doses. (XLSX 10 kb) In descending order, the number of times ("count") in which an agent at a particular concentration was identified in drug-dose combinations meeting filtering criteria. Drug-dose combinations with the same count are listed alphabetically. Rankings are determined separately for HMLE-shECad and HMLE-shGFP cells. See Supplementary Table 3

Supplementary Table 4: Combination indices. (XLSX) Combination Index (CI) values were calculated for AZD-7762 and obatoclax (Sheet 1) and prexasertib and venetoclax (Sheet 2) for CL cell lines BT549 and MDA.MB.436. CI values of greater than one indicate antagonism, of approximately equal to one indicate additivity, and of less than one indicate synergism. Growth Inhibitory (GI) values are listed for the indicated drug pairings. See Supplementary Table 4
